# Evolution of the Australian Institute of Sport Supplement Program over the last 25 Years

**DOI:** 10.1055/a-2891-6315

**Published:** 2026-07-13

**Authors:** Louise M. Burke, Gregory R. Cox, Ben Desbrow, Michelle Minehan, Aimee Morabito, Greg Shaw, Gary J. Slater

**Affiliations:** 1 Exercise and Nutrition Research Program 95359 Australian Catholic UniversityFitzroyVictoriaAustralia; 2 Faculty of Health Sciences and Medicine 3555 Bond UniversityCity of Gold CoastQueenslandAustralia; 3 High Performance UnitSwimming AustraliaBrisbaneQueenslandAustralia; 4 School of Allied Health, Sport and Social Work 97562 Griffith University Faculty of HealthGold CoastQueenslandAustralia; 5 Faculty of Health 2234 University of CanberraCanberraAustralian Capital TerritoryAustralia; 6 AIS Performance 453236 Australian Institute of SportBelconnenAustralian Capital TerritoryAustralia; 7 School of HealthUniversity of the Sunshine CoastMaroochydore DCQueenslandAustralia

**Keywords:** nutrition, behaviour change, ergogenic, inadvertent doping, sport

## Abstract

The Australian Institute of Sport Sports Supplement Program, launched in 2000, promotes evidence-based, safe, and legal supplement use in Australian high-performance sports. It has evolved into a comprehensive framework in response to regulatory changes, rapid growth in supplement products, and widespread access to both information and misinformation. Founded on a “food first” philosophy, it recognises circumstances where supplements may offer unique advantages, including addressing nutrient deficiencies, providing support pre-, during and post-exercise, and supporting performance. Its principles were shaped to counter fragmented regulation, aggressive marketing, and athletes' reliance on non-expert information sources. Central to the framework is a dynamic ABCD classification system, categorising supplements according to the strength of scientific evidence for their effectiveness, safety, and anti-doping risk. Key initiatives include web-based education resources, strategic supplement provision, applied research, and robust governance—especially batch testing and integrity processes—to reduce anti-doping rule violations. The framework now serves as a benchmark for national and international sporting bodies and has driven a cultural shift that places sports dietitians at the forefront of supplement guidance and enhances collaboration across performance support and medicine staff. Future priorities include deeper digital integration, adequate resourcing to remain contemporary, and broader athlete and stakeholder engagement to maintain rigorous, evidence-informed standards.

## Introduction

The Australian Institute of Sport (AIS) Sports Supplement Program was established in 2000 to assist Australian athletes toward evidence-based, safe, permitted supplement and sports food use. Twenty-five years later, we review the evolution of the Program into a framework that has been widely adopted across many high-performance sports systems. We focus on the associated learnings to consider future challenges and opportunities in an environment where misinformation is rampant. Although a “food first” approach underpins performance nutrition, there are contexts in which athletes may benefit from supplementation. 1​ These include: (1) when nutrients are difficult to obtain in sufficient quantities without excessive energy or dietary consumption; (2) when nutrients are abundant only in foods that athletes dislike or avoid; (3) when the nutrient content in an ergogenic food is variable; (4) when concentrated doses are needed to correct deficiencies/support immune tolerance; (5) when foods are impractical to consume around training/competition; and (6) when food safety is a concern. Unfortunately, the ill-informed use of supplements can compromise athlete well-being and integrity.

While contemporary expert statements exist, 1​ 2​ ​3​ the AIS Sports Supplement Program was the first to change from a risk averse perspective to embrace athlete and coach interests. Furthermore, it is the only international resource committed to reviewing the efficacy of supplements and sports foods on a recurrent basis. This paper reviews how the Program/framework has evolved to ensure that athletes have been provided with evidence-based information on supplements over the past quarter century.

## Background—Supplementation practices of Australian athletes in the 90s

Athletes and coaches have long pursued performance edges via supplements, often swayed by emotive marketing, peer testimonials, and brand prestige over scientific merits. The 1994 US Dietary Supplement Health and Education Act (Food and Drug Administration, 1994) lead to reduced supplement regulation, with global impact. 4​ Combined with the arrival of the internet, the 1990s signalled a new era of supplement availability. Indeed, the widespread use of dietary supplements within Australian sports was confirmed by a landmark investigation of the supplementation practices of Australian swimmers at the 1998 World Swimming Championships. Here, all but one athlete acknowledged the use of supplements in training and/or competition, with the majority reporting the use of two or more products. 5​ An expert panel convened to oversee the investigation made several recommendations, including

1. Greater collaboration between agencies responsible for supplement safety to enable athlete and coach education,
2. Development of an expert panel on supplement safety,
3. Consideration for an accredited supplement testing program, funded by manufacturers, and
4. Notification of anti-doping rule violations (ADRVs) associated with supplement consumption.

Contemporaneously, the AIS Department of Sports Nutrition led an internal review into the attitudes, knowledge and practices of athletes/coaches within its scholarship environment concerning supplements/sports foods. Key findings were

Changes in the international regulation of supplements created opportunities for new substances to be included in products, shifting the burden of proof of safety and efficacy away from manufacturers. 6​ The rapid increase in the number of supplements and individual ingredients created difficulties for AIS practitioners to keep pace with new products and claims. Athletes and coaches were usually unaware of the regulation of supplements and the strategies used by companies to circumvent these regulations.
There was misalignment between the position of the strict avoidance of supplements by antidoping agencies and the practices of athletes. Consequently, athletes relied on advice about the supplement use from outside their official performance support team. Research on supplements such as caffeine 7​ and creatine 8​ had confirmed performance benefits and protocols of use. Such information was at odds with official advice that supplements failed to enhance athletic performance.
The distinction between common and regulatory definitions of a “supplement” and “sports food” was becoming increasingly unclear, with many traditional sports foods including “ergogenic” ingredients.
The inflated rewards for successful performances and a recognition of the small margins of victory, 9​ provided incentives for athletes/coaches to consider any nutritional strategy.
The Internet/mail order/International travel enabled increased access to diversified supplement environments.
Supplements were commonly distributed through network marketing (i.e., independent distributors selling products to clients using testimonials).
The potential for the inadvertent ingestion of substances on the World Anti-Doping Agency (WADA) list of prohibited substances, with subsequent ADRVs, was confirmed by their presence in supplements. 10​
The number of supplement manufacturers/distributors 11​ increased the likelihood of endorsements resulting in free/subsidised access to products for athletes.

## Particular concerns related to the use of supplements by Australian Institute of Sport athletes

The lack of identified performance discipline with expertise and mandate to provide supplement advice to AIS athletes, created inconsistency in the delivery of support.
Athletes/coaches had reduced engagement with AIS dietitians due to the perceived lack of expertise or credibility associated with the official conservative view of sports supplements.
Factors demonstrated to make a major contribution to performance were potentially overlooked in favour of supplements. Unlike dietary strategies, the use of supplements was convenient and did not require extensive behaviour change.
Athletes were particularly interested in supplements that were marketed with emotive storytelling and other athlete endorsement, irrespective of scientific evidence.
Many athletes practiced an ad hoc approach to supplement use—including intermittent and/or concurrent use, limiting the opportunity to explore the efficacy of individual supplements.
An inability to source and/or document supplement use by AIS athletes.
Products used by AIS athletes implied “endorsement” which allowed products to flourish without scientific evidence of efficacy.
Dealing with the enquiries from independent distributors of network-marketed supplements was time-consuming for AIS dietitians.
The federally funded AIS represented a risk-averse environment requiring transparency over the investment of public funds.

## 2000–2012 Implementation of the AIS Sports Supplement Program

The AIS established an inter-disciplinary Sports Supplement Panel with daily management by the Department of Sports Nutrition. Key principles underpinning this program were considered novel and contradictory to conventional advice. For example, athletes and coaches would collaborate with sports dietitians to integrate evidence-based supplements and sports foods into individual performance nutrition plans. Supplements and sports foods could be practically managed via the use of a classification hierarchy that divided supplements into groups based on the strength of the evidence, with an appreciation of the risk of an ADRV. Specifically, four groups were identified (ABCD), with further stratification to identify strategies by which certain products could contribute to a performance nutrition plan. The categorisation of supplements and sports foods within these groups evolved with new knowledge and administration protocols. The original ABCD classification of supplements within the program was as follows:

**Approved or recommended for use by AIS athletes**
**Considered for provision to AIS athletes only under a research protocol**
**Supplements which have no proof of beneficial effects and are therefore not to be provided to official AIS programs**
**High risk/prohibited supplements should not be used by AIS athletes**

A cornerstone of this pragmatic approach to the classification of specific supplements included addressing the following three questions:

1. ***Is it safe?***
2. ***Is it effective?***
3. ***Is it permitted for use in sport?***

## 2013–2020 Evolution of the AIS Sports Supplement framework

A restructuring of the Australian high-performance sports system occurred following the London 2012 Olympic Games. The gradual devolution of the AIS removed its direct responsibility in the daily preparation of athletes. Meanwhile, it assumed a new leadership role, in conjunction with the state academies/institutes of sport (forming the National Institute [of Sport] Network or NIN), to develop technical expertise and resources in the Australian high-performance sports system. Accordingly, the AIS Sports Supplement Program shifted from an operational activity to a framework available to National Sporting Organisations (NSOs). NSOs and other organisations used the framework to develop bespoke sports supplement policies and/or programs that addressed their specific needs and resources, while complying with the National Integrity Framework. While the evolution of the classification system and education resources continued during 2013–2018 under the previous management structure within the AIS, a further restructuring in 2018 removed the key resources and personnel involved in these activities.

## 2021 Operational and resource updates

The contemporary AIS Sports Supplement Framework was relaunched in 2021, under new governance and expanded engagement with stakeholders. A Steering Committee now sets the strategic direction associated with regulatory partners, while a Framework Scientific Committee involving internal (AIS) and external experts manages the framework categorisation and education resources. Regular updates and reporting schedules continue to promote transparency and currency. The framework operates in collaboration with NSOs and other stakeholders to provide guidelines, resources and a forum to identify key projects that will enable NSOs to implement bespoke sports supplement policies, programs and guidelines, while meeting regulatory requirements. Key education resources are made freely accessible via the framework website to promote supplement literacy and transparency in the general sporting community. A current ABCD classification system taxonomy is presented in Fig. 1 , and Fig. 2 illustrates the decision tree summarising considerations related to the supplement use.

**Fig. 1:**
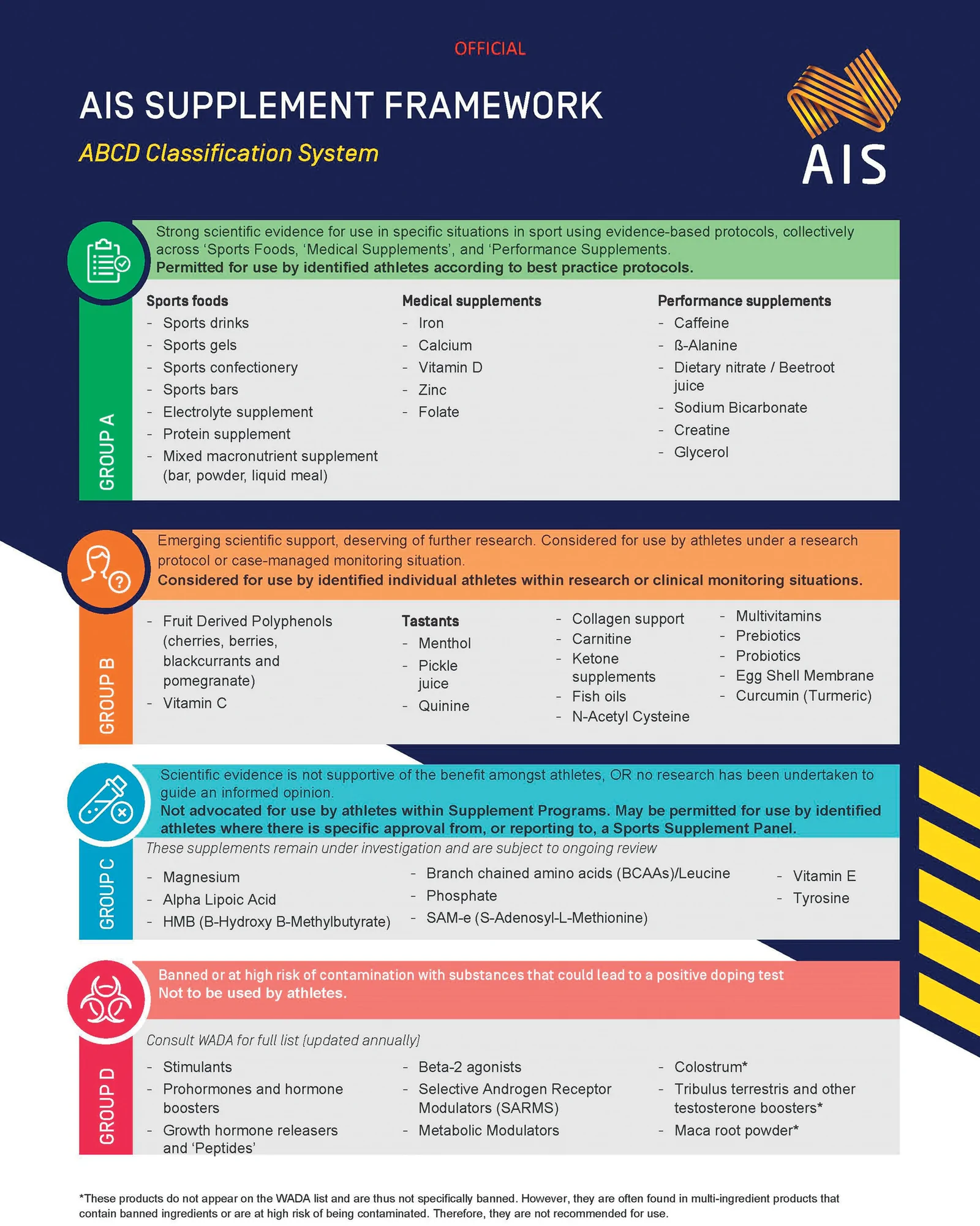
The contemporary AIS ABCD classification of supplements into one of four groups based on scientific evidence related to safety, efficacy and legality. 2025 classification of supplements according to the AIS ABCD Supplement Framework. Source: https://www.ausport.gov.au/ais/nutrition/supplements.

**Fig. 2:**
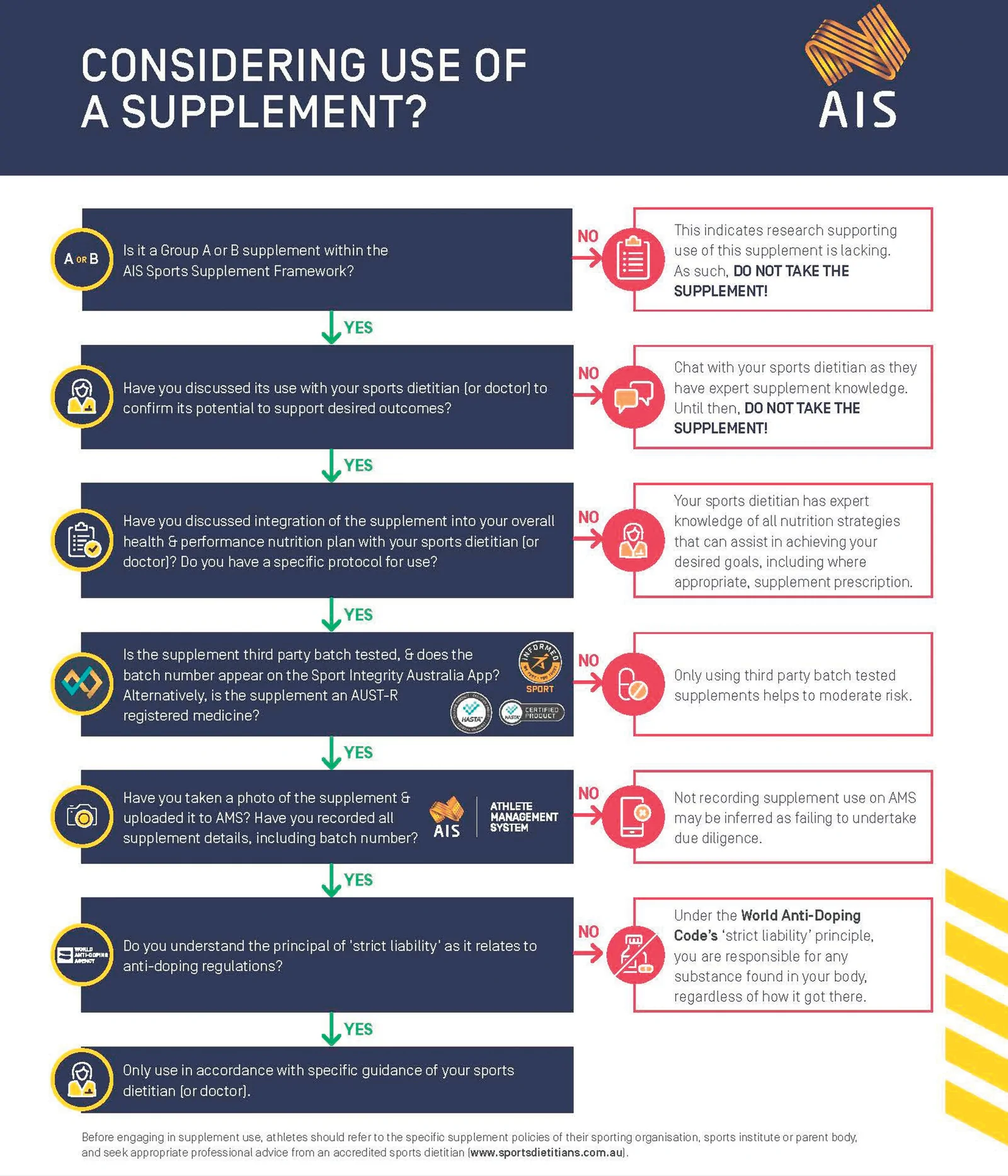
Decision tree on considerations related to supplement use within the context of the AIS Sports Supplement Framework. Group A supplement = strong scientific evidence for use under specific situations in sports using evidence-based protocols, collectively across ‘Sports Foods, ‘Medical Supplements’, and ‘Performance Supplements’; Group B supplement = emerging scientific support, deserving of further research; AUST-R = Australian Register of Therapeutic Goods Registered. Source: https://www.ausport.gov.au/ais/nutrition/supplements.

## Enabling supplement behaviour changes/reflections

Recognising that supplement risk-minimisation involves a myriad of determinants, behavioural frameworks (e.g., Capability, Opportunity, Motivation, Behaviour [COM-B] model) 12​ have been employed to evaluate the likelihood of strategies to reduce the risk of inadvertent ADRVs. 13​ The original AIS Sports Supplement Program placed emphasis on four pillars, which, on reflection, align to the recognised determinants of behaviour.

1. **Education of stakeholders (Capability/Motivation)**
2. **Strategic supplement provision (Opportunity)**
3. **The support of research and knowledge development (Capability/Motivation)**
4. **The critical role of governance (Opportunity)**

## Pillar 1/education (Capability/Motivation)

Athletes historically sought supplement guidance from coaches, family, peers, and self-discovery. 14​ In contrast, the AIS Sports Supplement Program placed education of scholarship athletes and coaches at its core. Initial physical resources, including a Sports Supplements Toolbox and fact sheets summarising priority Group A/B products, positioned sports dietitians as the professionals responsible for advice and education. The advent of digital platforms facilitated resource evolution and versatility while promoting sharing of intellectual properties across the broader sports community.

## Pillar 2/strategic supplement provision (Opportunity)

Initially, a range of Group A sports foods/supplements were provided to AIS scholarship athletes (2000–2012), according to protocols that considered risk-to-benefit analysis, the requirement to record provision/use and opportunities to observe their effectiveness in achieving health or performance goals. For example, low-risk products (e.g., sports drinks) were distributed to individual athletes or teams to manage independently. In contrast, “recovery bars” were set up in secure locations managed by a sports dietitian to provide targeted access to sports foods and “everyday” foods in the post-training/event environment. Medical supplements were dispensed only after a medical consultation, appropriate (re)testing of nutrient status and dietary counselling. Meanwhile, performance aids (e.g., caffeine/creatine) were dispensed as part of a personalised multi-disciplinary plan that considered the contextual appropriateness and athlete readiness. In all cases, provision was associated with reminders about athlete responsibility for decisions around supplement use, education around evidence-based protocols and procurement of products judged to be at low risk of causing an ADRV.

## Pillar 3/research and knowledge development (Capability/Motivation)

A recent bibliometric analysis ranked the AIS as the most prolific research institute for supplements in sport and exercise, 15​ a result underpinned by the outputs of the team led by Louise Burke (Head, AIS Nutrition Department for 30 y). Research productivity was supported by a performance environment that encouraged inter-disciplinary collaboration, while applied research was integrated into routine athlete performance support, ensuring that athletes and coaches were key stakeholders in decisions concerning supplements. AIS Science research provided an environment in which stakeholders were better able to quantify the outcomes of supplement use on variables of interest, including performance outcomes, health metrics and/or tolerance to various administration protocols. This information guided personalised prescription decisions, including specific strategies at key competitions. Such information was also critical in shaping the understanding of Group B supplements, which were characterised by emerging and/or conflicting research outputs.

Members of the AIS Sports Supplement Program were actively engaged in applied research on a range of supplements, including medical supplements, 16​ sports foods/drinks 17​ ​18​ and sports gels. 19​ Numerous investigations were conducted on performance supplements including caffeine, 20​ ​21​ creatine, 22​ ​23​ β-alanine 24​ and bicarbonate, 25​ ​26​ nitrate, 27​ 28​ ​29​ exogenous ketone supplements, 30​ ​31​ β-hydroxy β-methylbutyrate (HMB), 32​ pre-cooling aided by glycerol and/or sports drinks 33​ ​34​ and tastants such as quinine. 35​ Health supplements that were investigated included collagen 36​ 37​ ​38​ and probiotics. 39​ The potential synergistic effects of combining performance supplements 40​ 41​ ​42​ or repeating their use in events with multiple rounds 27​ was investigated, in acknowledgement of the challenges of real-world applications of supplement use. 43​ Some of these projects were achieved within PhD programs embedded at the AIS with the support of academic partners. Furthermore, some studies were implemented using performance protocols that specifically simulated the events of interest at the Olympic Games or World Championships. 30​ 33​​ ​34​ ​40​ Beyond empirical evidence, the AIS generated guidance on broader issues associated with supplements, including ADRV risk 5​ ​44​ and research designs for investigating the ergogenic potential of supplements. 45​

## Pillar 4/governance (Opportunity)

The absence of commercial third-party supplement testing before 2004 required early activities of the AIS Sports Supplement Program to take a pragmatic risk management approach to the growing awareness of evidence of ADRV risks associated with supplement use. 10​ Typically, this involved collaboration with suppliers to understand sources of ingredients and manufacturing processes. In the period preceeding commercial third-party supplement testing, the AIS worked with the Australian Sports Anti-Doping Authority (now, Sports Integrity Australia) and the Australian Sports Drug Testing Laboratory to set up a program offering this service to Australian manufacturers to assess the prevalence of WADA prohibited substances in products on the Australian market. The AIS Sports Supplement Program required the documentation of performance supplement purchases and dispensation within its research projects and individual programs. The foresight and value of such activities, promoting transparency and accountability around supplement use, were proven to be valuable following the 2013 Australian Crime Commission report entitled, “Organised Crime and Drugs in Sport.” 46​ This report identified the problematic use of banned substances in sport under the guise of “Supplement Programs” in professional sports. The AIS Sports Supplement Framework was subsequently promoted as an exemplar for supplement management.

Contemporary NIN and NSOs practices for Olympic and Paralympic sports incorporate the AIS Sports Supplement Framework to govern their supplement programs and policies. The current AIS Sports Supplement Framework advocates that athletes use only third-party batch-tested supplements that are documented on an athlete management system (AMS), upholding the World Anti-Doping Agency (WADA) due care principle. A recent Australian investigation has reinforced the need for athletes to remain vigilant, with one-third of 200 supplements purchased online within the Australian marketplace contained WADA-prohibited substances. 47​ Indeed, between 2016 and 2019, one-third of ADRVs in Australia were attributed to sports supplements, a dramatic increase over the previous decade. 48​

## Evolution of the AIS Sports Supplement framework—classification of specific supplements

The placement of supplements and sports foods within the ABCD classification system is dynamic, changing in response to emerging knowledge about their safety, efficacy and legality. A founding principle of the classification system was to focus on products with the greatest likelihood of providing benefits to athlete health and performance. Therefore, Groups A and B have received the majority of attention, with research and education activities continuing to evolve the placement of products and ingredients within these categories as well as the information provided about their potential uses in sports. Indeed, while the original version included the names of ingredients of Group C products, later iterations removed these to reduce interest in the group. Instead, this group continues to specifically identify only those products which have been reclassified from Group A or B, as an additional reinforcement that the most recent evidence indicates minimal ergogenic benefits. Accompanying statements such as “if you can’t find the ingredient of your interest in Groups A, B and D, it probably belongs to Group C” serve to highlight the lack of priority afforded to these products. The special distinction of Group D products reminds athletes that some supplements may contain compounds included on the WADA Prohibited List. This group also contains products which, although not specifically named on the List, have been associated with controversy or confusion regarding their permitted status. This includes maca root, which has been associated with a false positive test for raised testosterone attributed to analytical interference. 49​ Other purported “pro-hormone” products are also included in Group D. For example, *Tribulus terrestris* (marketed to elevate testosterone), is not banned per se however, is considered at a high risk of contamination and therefore advised against. Colostrum received placement in Group D in response to WADA concerns over its influence on plasma IGF-1 levels, despite evidence to the contrary. 50​

Fig. 3 provides an overview of changes in Groups A and B supplement classification across Summer Olympic Games cycles since the Program inception in 2000. The classification of Group A Sports Foods has remained relatively stable across the lifespan of the framework reflecting the historical, 51​ multifactorial 52​ and ever evolving 53​ nature of the evidence for the benefits of consuming carbohydrates during many types of exercise. A more recent addition to Group A is protein supplements representing the appreciation of the benefits of the strategic intake of protein over the day and around exercise. 54​ Likewise, creatine, bicarbonate and caffeine have been classified as Group A Performance supplements since 2000, reflecting both the early 55​ 56​ ​57​ and continued 58​ 59​ ​60​ evidence to support their targeted use as performance aids. Glycerol was also classified as a Group A Performance supplement in the initial iteration of the AIS Sports Supplement program but reclassified to Group D, when it was added to the WADA list as a prohibited masking agent (2010–2018). Following its removal from the WADA list and the continuation of studies of its effect as a hyperhydrating agent to assist endurance performance under hot weather conditions, 61​ glycerol was reinstated to Group A.

**Fig. 3:**
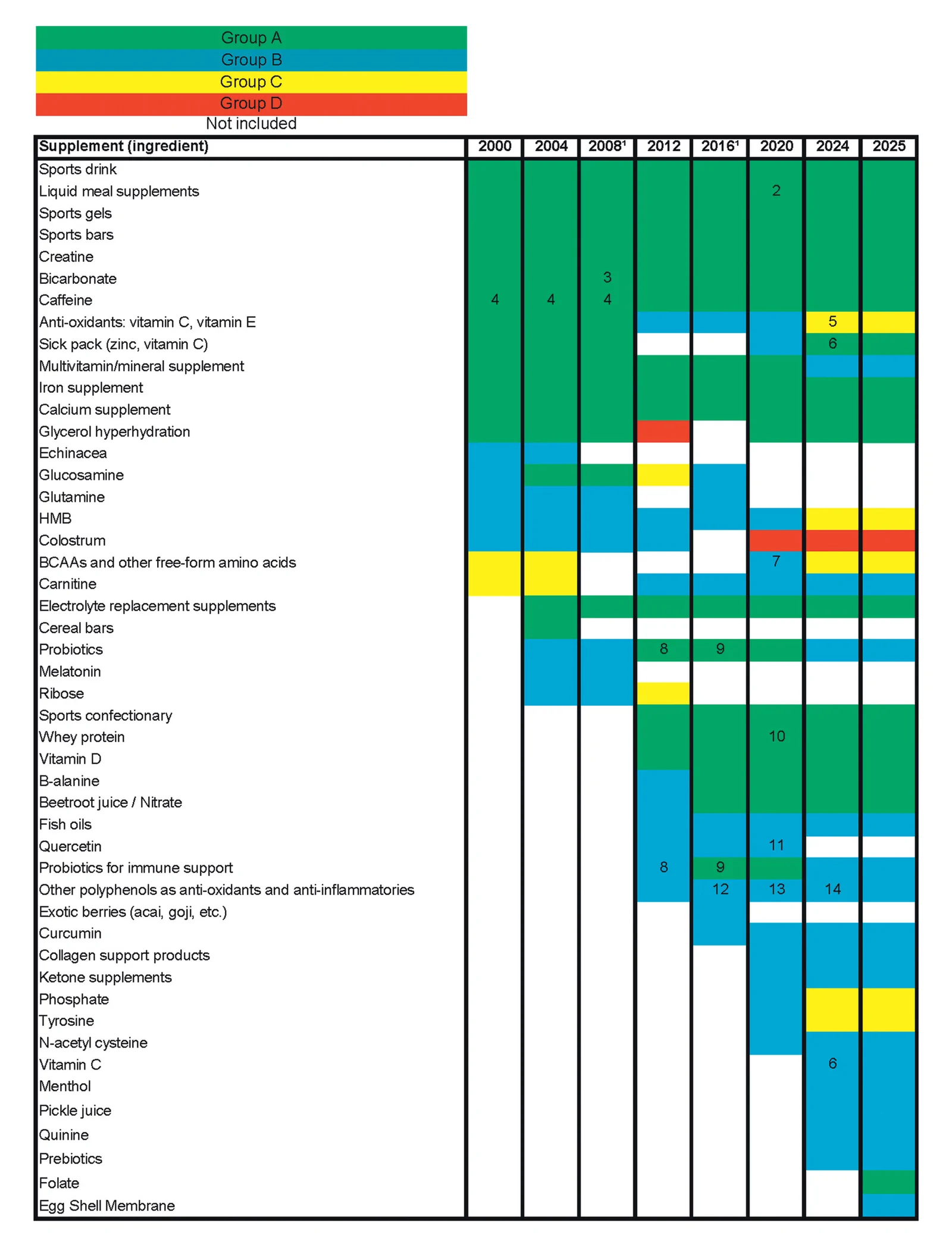
Evolution of the AIS Sports Supplement Framework over the last 25 years, including all supplements that have been classified as either Group A and/or B during that time. ^1^ No Group C or D supplements were documented in 2008 and 2016. ^2^ Renamed to ‘Mixed Macronutrient Supplement (bar, powder, liquid meal) from 2020. ^3^ Included Citrate in 2008 but reverted to bicarbonate in 2012. ^4^ Caffeine for use under special consideration only in 2000–2008. ^5^ Became vitamin E only in 2024. ^6^ Sick pack separated into zinc (Group A) and vitamin C (Group B) in 2024. ^7^ Became BCAAs/leucine in 2020. ^8^ Probiotics separated into gut protection (Group A) and immune support (Group B) in 2012. ^9^ Probiotics were combined for gut health and immunity in 2016. ^10^ Renamed to Isolated Protein Supplement from 2020. ^11^ Renamed to quercetin, EGCC, epicatechins and others in 2020. ^12^ Renamed to Tart cherry juice in 2016. ^13^ Renamed to cherries, berries and blackcurrants in 2020. ^14^ Renamed to fruit derived polyphenols in 2024. BCAAs, branched chain amino acids; HMB, U+03B2F;-hydroxy U+03B2F;-methylbutyrate.

Given the intentional focus on products with emerging and/or mixed scientific support, Group B represents the most pliable tier within the ABCD classification system. Indeed, its original goal within the Program (2000–2012) was to promote active research within the AIS system on new products or products with the potential to be strategically used for high priority events or athletes. This was particularly evident during the 2009–2012 Olympiad when Group B status was conferred to nitrate and β-alanine to direct targeted research activities to these new products. Although research continued to occur with more specialised uses of Group A products (e.g. sex differences in response to caffeine in the London Olympic Games Road Cycling Time Trial 40​ ), the deliberate retention of nitrate and β-alanine in this category, even as positive evidence accrued, served an institutional priority as much as an objective reflection of the literature.

The later transition from the single-institution focussed Supplement Program into a broader Supplement Framework reduced the opportunity for the assignment of products into Group B to be “politically” determined. Currently, decisions regarding the Framework Scientific Committee’s classification of products or ingredients reflect representation from stakeholders (NSOs and NIN) as well as objective metrics around their effectiveness, safety, and permissibility. Ideally, classification to Group B is a temporary position as evidence accrues for reassignment to either Groups A and C. For example, ketone ester supplements, phosphate, tyrosine and *N* -acetyl cysteine (NAC) were all added to Group B in 2020. However, only ketone esters and NAC remained in the 2026 category due to the sustained interest of stakeholders, and continued research outcomes deemed to be supportive without achieving definitive results. 62​ ​63​ In contrast, phosphate and tyrosine have been reclassified to Group C, reflecting the lack of interest among athletes and limited recent research into the ergogenic potential of these supplements. 64​ ​65​ The duration of Group B classification is not defined. For example, HMB retained a long-term status as Group B due to high level athlete interest, but was eventually declassified to Group C when a definitive meta-analysis concluded that HMB did not affect strength and body composition in trained and competitive athletes. 66​

## Impact of supplement program/framework

To date, only one study has directly assessed the impact of the AIS Sports Supplement Program on supplementation behaviours of athletes. 67​ The investigation examined AIS swimmers over an 11-year period spanning pre- and post-implementation of the program. Although the overall supplement use remained similar, swimmers in the AIS environment used more evidence-based products and fewer low-efficacy/high-risk supplements than a comparable non-AIS cohort. 68​ Internationally, the AIS Sports Supplement Framework has been used as a benchmark to assess athlete supplementation practices in national level samples 69​ and across sports, including sailing, 70​ cycling, 71​ 72​ ​73​ football, 74​ ​75​ swimming, 76​ ​77​ running, 78​ ​79​ triathlon, 80​ fencing, 81​ and Handball, 82​ ​83​ plus referees. 84​ This highlights the framework’s utility as a categorisation tool and its broad recognition and credibility.

Digital analysis of the AIS Sports Supplement Framework website provides further insights into its reach and use. Fig. 4 summarises 2024 access metrics, including total users, sessions, most visited pages and device used for access. Page hits across the ABCD Framework mirror the relative value of supplement categories, with Group A pages receiving the most traffic. While the AIS framework remains popular in Australia (52.8 K sessions), it was mostly engaged by viewers within the United States of America (102.6 K sessions) and Europe (66.5 K sessions), confirming its international appeal.

**Fig. 4:**
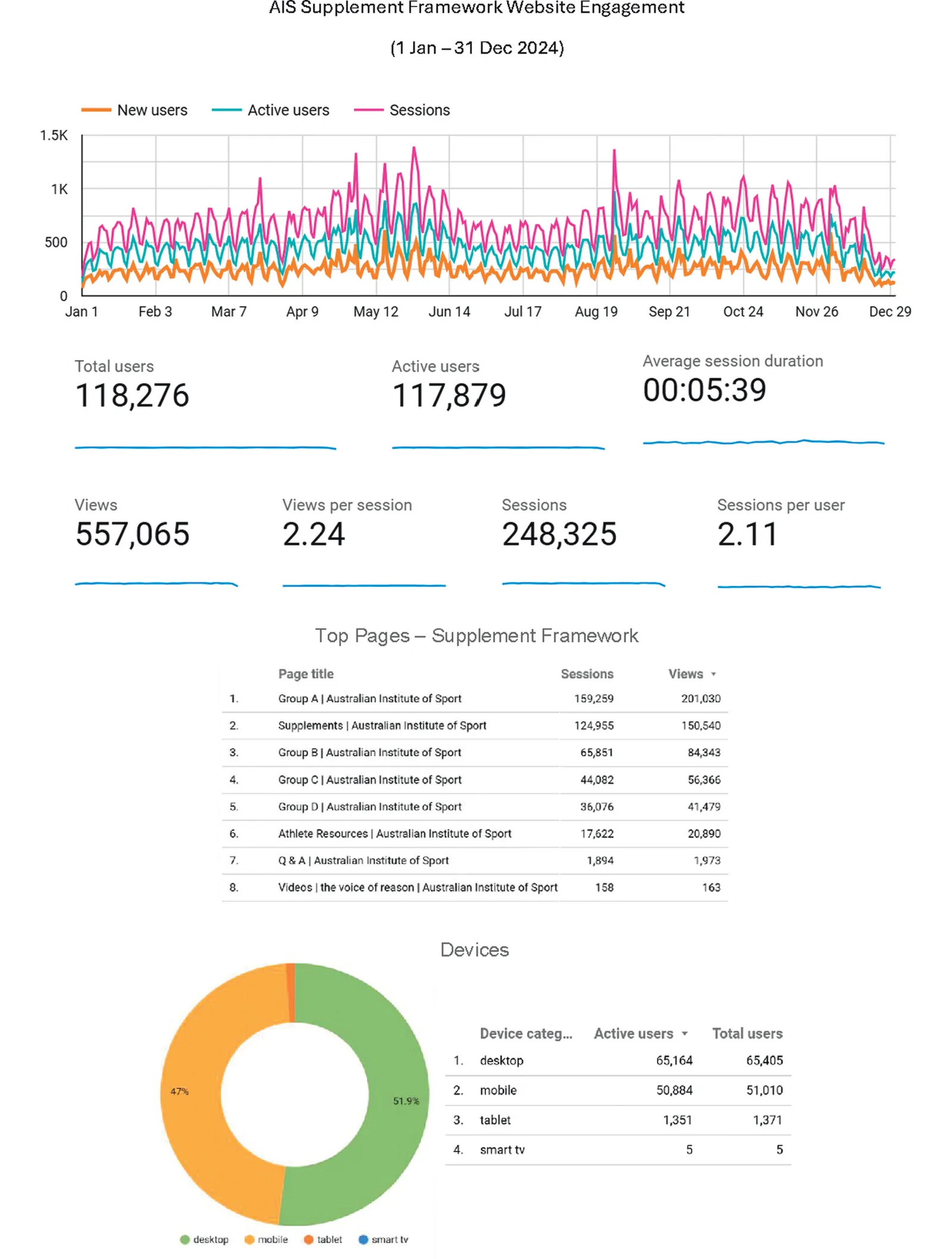
AIS Sports Supplement Framework website metrics (1 Jan–31 Dec 2024), including total users, page views and average session duration. Sourced via Google Analytics. ASC, Australian Sports Commission.

## Future challenges and opportunities

### Education

The key goal of the AIS Sports Supplement Framework is to enable sports dietitians, athletes, and other stakeholders to make informed decisions. To remain relevant, the framework must provide value to practitioners while accommodating athletes’ diverse learning preferences. Athletes value individual engagement with credible, sport-specific performance nutrition practitioners, 85​ alongside accessible, reputable online information. 86​ Currently, the framework offers largely static digital resources. Social media provides dynamic education opportunities, is widely used by athletes 87​ and is increasingly adopted by performance nutrition practitioners. 88​ Its acceptance among nutrition professionals 89​ enables visually engaging, practical content through athlete-preferred mediums. 90​ Although athletes remain sceptical of online and social media nutrition information, 87​ ​90​ the framework’s independent credibility could overcome this. Strategic collaboration with the framework aligned athlete influencers could further boost engagement. 91​ Novel use of knowledge repositories and emerging large language model and agentic capabilities could transform the original static evidence folders into a more contemporary, world leading knowledge bank. This would maintain the framework’s education purpose, while leverging technology to influence contemporary supplement behaviours as originally intended.

### Environment

Manipulating food access within the daily performance environment influences athlete dietary intake. 92​ While a food-first approach is preferred for athlete’s overall nutrition goals, practical challenges often arise with perishable foods requiring transport, storage or preparation and/or tolerance and absorption constraints tied to athlete lifestyle or exercise commitments. Strategic availability of targeted sports foods in the sporting environment offers a convenient solution to meet acute nutrient needs before, during and/or after exercise. For example, providing sports drinks or mixed macronutrient drinks at event finish lines increases post-exercise recovery intake without compensation at a subsequent meal. 93​ The AIS Sports Supplement Framework evaluation suggested provision of evidence-based supplements and sports foods in the AIS training environments enabled behaviours associated with the strategic use of these products, with the concomitant reduced use of Group C and D products. 67​ Similar effects of supplement provision on compliance have been observed in clinical populations 94​ and may be particularly effective when financial issues contribute to the gap between knowledge and practice. The optimal behaviour change typically pairs product availability with education, personalised prescription, follow-up, and trusting practitioner relationships grounded in evidence. 95​

While provision of batch-tested supplements is resource-intensive, it warrants consideration. Supplement provision was enabled at the AIS through strategic collaboration with respected industry partners, who shared a similar philosophy of supporting the use of safe and effective products. These shared goals and knowledge exchange are characteristic of other successful nutrition industry collaborations. 96​ Given that available resources may be a limitation to influencing supplement provision in the daily performance environment, a range of solutions is proposed below to enable access to evidence-based supplements and sports foods facilitating access to third party batch tested supplements at a discounted rate through an online company that specialises in retailing only third-party batch-tested supplements. Individuals or small teams do not have the capacity to organise specialised testing of the entire production run of a desired sports supplement or food. By organising to purchase and distribute batch tested products from a range of manufacturers, such a company address a niche but valuable need and increases tincentive for manufacturers to undertake testing without having to hold inventory. Although this option places the financial burden on the athlete, it increases their access to safe supplements at some discount. Centralising the purchase point to a single company simplifies purchasing and minimise opportunities for athletes to be distracted by other online products or accidentally purchase non-tested products
Assembling a collection of (batch-tested) individual samples from supplement manufacturers to allow athletes to trial various products from Group A or B before committing to purchases. Ideally, this is done with the guidance of a performance nutrition practitioner.
Arranging partnership(s) with reputable supplement manufacturers to provide discounted access to batch-tested supplements according to practitioner recommendations. The sponsorship arrangement may reduce the financial burden on the athlete but may also reduce the range of products according to the type and number of commercial partnerships.
Forming partnerships with reputable supplement manufacturers to provide batch-tested supplements and sports foods for management within a sports environment. Access might range from self-management of low-risk products at the point of use (e.g. sports drinks at a training venue) through to supervised interaction with a performance nutrition practitioner. If access to the full range of desired products is not available, athletes may be able to purchase additional products at discount or contribute a subsidy to the sports program.
Establishing fully funded programs that purchase all supplement and sports food needs for their athletes from batch-tested supplies, and without the encumbrance of sponsorship requirements or demands. Establishing fully funded or sponsor-partnered models which accommodates a research program as well as supplement provision. May lead to the development of bespoke products for special needs or individual athletes and could be partnered with academic institutions to support research activities.

### Research and knowledge development

Ongoing research into the efficacy of supplements among specific athletic populations is supported by both the AIS Sports Supplement Steering Committee and Framework Scientific Committee, yet committee members have limited capacity to directly undertake applied research on sports supplements. As such, the Framework Scientific Committee should explore external collaborations, particularly with academic institutions that are resourced to support applied research and can co-design investigations with performance support practitioners, athletes and coaches. Strategic partnerships between industry and academic institutions have already developed novel products such as slow release β-alanine supplements 97​ and specialised formulae and delivery mechanisms for higher carbohydrate sports drinks. 98​ 99​ ​100​ Although industry funded research can attract prejudice, limited access to alternative grants for sports performance research, often makes such partnerships a necessity. When managed professionally, with independence and scientific rigor, such research can benefit all contributors.

Performance nutrition practitioners should seek opportunities within their organisations to support research and build research capacity. In its simplest form, practitioners could prioritise controlled case studies to generate insights into supplement effectiveness in individual athletes. Developing evidence-based, personalised protocols for specific applications is valuable for all supplements, 101​ but is particularly important when guiding prescription of Group B supplements. Capturing this information centrally on an AMS would allow subsequent analysis, providing insights from multiple individual trials that may inform future supplement classification decisions.

Considerations for supplement classification or reclassification are heavily influenced by performance nutrition practitioners within the high-performance system, alongside the framework’s identified international experts. While this has ensured the AIS Sports Supplement Framework classification is aligned with other groups which categorise sports supplement efficacy amongst athletes, 2​ ​102​ it fails to acknowledge other approaches. For example, there is some evidence to suggest the collective opinion of a diverse and independent group may be superior to that of a single expert, a method known as wisdom of the crowd, 103​ which is being explored in sports. 104​ Although some wisdom-of-the-crowd principles are used to support the annual review of supplements for reclassification, this does not continue once an expert is engaged, apart from the input from Framework Scientific Committee members. Consensus meetings and subsequent statements are fundamental to expert driven guidelines, 105​ yet they are resource intensive, which may help explain why the most recent example focussed on dietary supplements remains the 2018 IOC Position Statement. 2​ Remote connection now offers a viable option for connecting experts internationally and should be considered where it offers benefit for all parties.

The AIS Sports Supplement Framework Scientific Committees have resisted transition to a more structured scaffolding with explicit classification criteria for the study number and quality in categorising supplements into ABCD groups. However, the Framework Scientific Committee does acknowledge the excellent methodological guidance available elsewhere for investigating sports supplement efficacy. 3​ ​45​

### Situational efficacy

Situational efficacy of supplements recognises that a supplement may have efficacy and therefore be classified as Group A or B in one sporting context but not another. For example, sodium bicarbonate, a Group A Performance Supplement, has strong evidence of performance benefit in events requiring muscular endurance rather than strength. 106​ Indeed, attempts have been made to categorise supplements as green (strong supporting evidence of efficacy), amber (moderate or emerging evidence) or red (lack of evidence, high risk of contamination and/or currently prohibited in sport) according to endurance or strength/size outcomes or impact on health. 102​ This remains a challenge for the AIS Sports Supplement Framework, highlighting the critical role of the sports nutrition practitioner in inferring potential efficacy of a supplement as part of an athlete’s overall performance nutrition strategy.

The ABCD classification evaluates individual supplements and sports foods based on evidence of efficacy, safety, and legality, but does not currently account for potential interactions when multiple supplements are co-ingested. This limitation applies irrespective of the interaction direction: synergistic or antagonistic. For example, β-alanine and sodium bicarbonate may provide additive benefits on high-intensity performance when combined, potentially exceeding individual effects, 107​ although evidence is heterogeneous. Conversely, co-ingestion of calcium with iron can markedly reduce iron absorption by 28–62%, blunting iron's efficacy. 108​ Future iterations of the Framework could incorporate interaction data where robust evidence exists.

### Collaboration with other stakeholders

In many areas of life, binary approaches such as good/bad or right/wrong provides simplicity and clarity, and when safety or health concerns are involved, a cautious or conservative choice is generally assumed. It is therefore understandable that sporting authorities, including National Governing Bodies, Anti-Doping Agencies and Sports Medicine/Science Authorities, have traditionally advised athletes against supplement use. Since there is no practical way to eliminate doping risk from the use of supplements and sports foods, this is likely to remain the official policy of anti-doping agencies. However, over the past decade, other authorities have increasingly recognised that advice regarding the use of supplements and sports foods is complex and warrants a more flexible, pragmatic approach. The AIS Sports Supplement Framework illustrates some of this complexity, including the need to engage with athletes and coaches who already use supplements, acknowledge broader community use, and recognise situations in which sports foods, nutrient-based supplements and performance supplements can contribute to the athlete’s performance nutrition plan and outcomes. 1​ This shift has supported education campaigns that promote considered supplement use within a performance nutrition plan, with agencies such as the International Olympic Committee adopting an approach first popularised by the AIS Sports Supplement Program. 2​

Future projects should continue to engage with Australia’s regulatory bodies on supplements and sports foods, supporting efforts to reduce ADRVs by improving access to evidence-based, batch tested products. Although challenges will remain concerning the safe and effective use of supplements and sports foods within Australia’s high-performance sports system, collaboration among stakeholders around the framework will continue to facilitate pragmatic solutions that optimise performance, promote athlete health and maintain fairness.

## Conclusions

The AIS Sports Supplement Program and subsequent framework provide a case study in managing the complex issue of supplement use in high-performance sport through evidence-based education, provision, research, and governance. Over more than 25 years, the program and framework have demonstrated the value of a dynamic, evidence-driven approach that can adapt to changes in sports science knowledge, the marketplace, regulatory systems and the roles of performance support personnel. The integration of the ABCD classification system with robust education platforms and supplement provision protocols has promoted safer, more effective, and legally compliant supplement among Australian athletes, while fostering shared responsibility and transparency.

Programmatic evolution from direct product provision to the development of resources and frameworks accessible to all stakeholders have extended the AIS impact nationally and internationally, offering a model for supplement policy in sport. Ongoing collaboration with sporting organizations and regulatory bodies, regular updates to classification and educational resources, and continued commitment to best-practice governance will be essential to keeping the model current and adaptable. Although challenges remain, including information overload, aggressive marketing, and anti-doping concerns, the AIS Sports Supplement Framework demonstrates that inter-disciplinary, evidence-based approaches can support athlete safety and performance while minimizing risks. Future progress will depend on stronger research partnerships, new education technologies, transparency, and adequate funding and resourcing. The principles underpinning the framework continue to support ethical, safe and effective supplement guidance for athletes.

The authors hope that authorities funding and supporting sport in Australia and internationally recognise the value of the AIS Sports Supplement Framework. Designed to keep Australian athletes at the forefront of sports nutrition practice, it has become an international benchmark and resource, promoting change in the discourse and use of supplements and sports foods across the globe for elite to recreational athletes. It has also stimulated innovation in behaviour modification, performance research and education. Although supplements are sometimes viewed as peripheral or negative in sports, their role and the interest of athletes and coaches are legitimate. The sports nutrition and science community should continue to prioritise supplement education to promote safe and effective use. The AIS Sports Supplement Framework has led the way for 25 years, and its enduring principles continue to set the benchmark for integrity, safety, and innovation in supplementation practice.
